# Evaluation of the Efficacy of Low-Concentration Gaseous Chlorine Dioxide in Inactivating Airborne H5 High Pathogenicity Avian Influenza Virus in Vivo Model

**DOI:** 10.1007/s12560-026-09677-3

**Published:** 2026-01-23

**Authors:** Yik Lim Hew, Norikazu Isoda, Takanori Miura, Takahiro Hiono, Yoshihiro Sakoda

**Affiliations:** 1 Laboratory of Microbiology, Department of Disease Control, Faculty of Veterinary Medicine, Hokkaido University, Kita 18, Nishi 9, Kita-Ku, Sapporo, Hokkaido 060-0818 Japan; 2 International Collaboration Unit, International Institute for Zoonosis Control, Hokkaido University, Kita 20, Nishi 10, Kita-Ku, Sapporo, Hokkaido 001-0020 Japan; 3 Hokkaido University Institute for Vaccine Research and Development (HU-IVReD), Hokkaido University, Sapporo, Japan; 4 One Health Research Center, Hokkaido University, Sapporo, Hokkaido 060-0818 Japan; 5 Department of Molecular Biochemistry and Clinical Investigation, Osaka University Graduate School of Medicine, 1-7 Yamadaoka, Suita, Osaka 565-0871 Japan

**Keywords:** H5 HPAIV, Gaseous ClO_2_, Poultry, Disinfectants, TCID_50_, Chick model

## Abstract

H5 high pathogenicity avian influenza virus (HPAIV) continues to spread globally, causing several high pathogenicity avian influenza (HPAI) outbreaks in poultry and significant economic losses. Biosecurity measures that prevent the introduction of HPAIV represent a top priority for controlling HPAI outbreaks on poultry farms. Although these measures are crucial for minimizing HPAI introduction, outbreaks of viral infection on poultry farms persist, underscoring the importance of continuously improving biosecurity protocols. Therefore, safe and effective microbicide disinfectants could play an essential role in reducing viral spread by inactivating viral particles on surfaces and in the air. This study assessed the efficacy of gaseous chlorine dioxide (ClO_2_) against H5 HPAIV under both gaseous ClO_2_ inactivation setting and in vivo conditions. In the gaseous ClO_2_ inactivation setting, only low virus titers were recovered (< 0.5–1.5 log_10_ TCID_50_/mL) when H5 HPAIV aerosols were exposed to gaseous ClO_2_ (0.05 ppmv, 0.14 mg/m^3^) for 5 min, corresponding to an approximately 2.0–3.0 log_10_ reduction. Furthermore, in vivo, all chicks exposed to aerosolized H5 HPAIV, which were treated with 0.1 ppmv gaseous ClO_2,_ survived for 14 days post-challenge, demonstrating complete protection against the virus. The minimum effective concentration of gaseous ClO_2_ was 0.01 ppmv for 5 min of inactivation in the inactivation setting, and 0.05 ppmv for 5 min in vivo, indicating that relatively low concentrations are sufficient for effective viral inactivation. Therefore, gaseous ClO_2_ was effective at inactivating aerosolized H5 HPAIV and has potential for use as a disinfectant to prevent HPAIV introduction into poultry. (245/250) words.

## Introduction

Avian influenza is a highly contagious respiratory disease in poultry caused by influenza A viruses (IAV) (Howley et al., 2021). Wild migratory ducks act as primary reservoirs of avian influenza viruses (AIVs), enabling transmission among wild aquatic birds and various bird species. The clinical outcomes of AIV infection range from asymptomatic to severe, including fatal cases. AIVs from the genus *Alphainfluenzavirus* (family *Orthomyxoviridae*) comprise eight single-stranded, negative-sense RNA segments. Based on the antigenic characteristics of their surface proteins, they are categorized into 16 hemagglutinin (HA; H1−H16) and 9 neuraminidase (N1−N9) subtypes. AIVs are further classified as low pathogenicity (LPAIVs) and high pathogenicity AIVs (HPAIVs). The latter are restricted to H5 and H7 subtypes because of the presence of multiple basic amino acid residues at the cleavage site of the HA protein, which permits systemic infection in birds (Alexander, 2007; Dhingra et al., 2018; Escalera-Zamudio et al., 2020; Fouchier et al., 2005). The emergence of H5 HPAIVs from the A/goose/Guangdong/1/1996 lineage has led to diversification into multiple clades, which continue to threaten wild birds and poultry globally. H5 HPAIVs from clade 2.3.4.4b have been consistently isolated in Asia and Europe since 2016 (Lycett et al., 2019; Pohlmann et al., 2022), followed by rapid intercontinental spread to North and South America in 2022 and most recently to Antarctica (Banyard et al., 2024; Kandeil et al., 2023; Ruiz-Saenz et al., 2023). The global circulation of this clade has led to significant economic losses because of outbreaks in the poultry industry.

The introduction of HPAIV to poultry farms was believed to result from exposure to virus-contaminated excretions or secretions from wild birds. This process is facilitated by the movement of people, contaminated equipment, and vehicles (Koch & Elbers, 2006). In addition, the detection of wild waterbird-derived DNA in the atmosphere of farms demonstrated that the airborne dispersal of HPAIV might contribute to introducing viruses into the flock (Bossers et al., 2024). Therefore, biosecurity measures remain a top priority for controlling high pathogenicity avian influenza (HPAI) outbreaks on poultry farms. Segregation, cleaning, and disinfection are fundamental biosecurity principles that prevent the intrusion of people, wild animals (such as rodents or crows, which interact with both wild birds and poultry), or vehicles that can carry pathogens into the farm. Additionally, disinfectants that inactivate pathogens on farm surfaces and equipment can reduce the risk of HPAI outbreaks in poultry (Ministry of Agriculture, Forestry and Fisheries of Japan, 2025). Although current biosecurity measures are considered crucial in minimizing HPAI introduction, outbreaks in poultry farms have been continuously reported, underscoring the need to continually enhance these measures, including the adaptation of new technologies, as H5 HPAIV can be introduced through aerosolized contaminants from infected birds directly, through other animals, or by human activities. Safe and effective disinfectants are additional options to reduce the level of contamination by pathogens, and they are applicable to poultry farms, markets, and their surrounding environments. Disinfectants such as sodium hypochlorite (NaOCl) and quaternary ammonium compounds (QACs) are commonly used in poultry farms. NaOCl is known for its broad-spectrum disinfectant properties, as it effectively eliminates a variety of viruses, including feline calicivirus, human influenza virus, and measles virus, as well as bacteria such as *Pseudomonas aeruginosa* and *Acinetobacter* spp. (Köhler et al., 2018; Sanekata et al., 2010). In conjunction with QACs, NaOCl has also displayed significant virucidal activity against severe acute respiratory syndrome coronavirus 2 (SARS-CoV-2) and H7N1 LPAIV (Caschera et al., 2022; Ito et al., 2018). However, NaOCl is less effective in the presence of organic matter, and this compound can corrode the metal surface (Da Nizer et al., 2020; Han-Hsing Lin et al., 2021). Similarly, QACs lose virucidal properties under these conditions and at low temperatures (Kabir et al., 2021). Therefore, another disinfectant, such as chlorine dioxide (ClO_2_), might be considered to improve biosecurity on poultry farms.

ClO_2_ is a yellow, water-soluble gas with a characteristic chlorine-like odor and strong oxidizing properties. It is classified as an A-1 level disinfectant and recognized by the World Health Organization as safe and effective, and it is commonly used in commercial settings (Moran et al., 1953; Tao et al., 2024). Additionally, ClO_2_ is typically less corrosive to metals during disinfection-level exposure, making it more suitable for environments with sensitive equipment (Yue et al., 2024). Several studies have demonstrated the wide range of antimicrobial activities of liquid and gaseous ClO_2_ against bacteria, fungi, and viruses, including *Staphylococcus aureus*, *Penicillium chrysogenum*, SARS-CoV-2, and H7N1 LPAIV (Imoto et al., 2025; Kadota et al., 2023; Morino et al., 2011; Wilson et al., 2005). Numerous studies found that gaseous ClO_2_ exhibits virucidal efficacy against several influenza viruses, including H1N1 seasonal influenza in a mouse model [0.03 ppmv (0.084 mg/m^3^) after 15 min] and H7N9 HPAIV in vitro (5.0 ppmv after 1 h) (Ogata & Shibata, 2008; Sun et al., 2022). The antimicrobial effects of ClO_2_ are likely associated with the peroxidation of viral lipids, thereby disrupting the lipid envelope and protein shell (Ge et al., 2021). This capability has been demonstrated through the ability of ClO_2_ to inactivate influenza viruses by oxidizing the tryptophan residue (W153) to *N*-formylkynurenine in the HA protein, which is crucial for viral attachment to host cell receptors (Ogata, 2008). Despite the recognized broad-spectrum antimicrobial properties of ClO_2_, the virucidal efficacy of gaseous ClO_2_ against H5 HPAIV has not been thoroughly investigated in either in vitro or in vivo settings. Therefore, this study represents the first comprehensive investigation of the efficacy of gaseous ClO_2_ against H5 HPAIV using a chick model, providing novel empirical data to support its application. The obtained data will be applied to additional options in biosecurity measures to reduce HPAI risks on poultry farms.

## Materials and Methods

### Virus Preparation

An H5N1 HPAIV A/white-tailed eagle/Hokkaido/22-RU-WTE-2/2022 strain (WTE/Hok/R22/22; H5N1) previously isolated from a deceased white-tailed eagle (*Haliaeetus albicilla*) (Isoda et al., 2022) was propagated into 10-day-old embryonated eggs. Following virus propagation in allantoic fluid as confirmed by the HA assay (Yamamoto et al., 2011), the collected allantoic fluid was used as the viral stock. The final titer of the viral stock was determined as 10^9.0^ 50% tissue culture infectious dose (TCID_50_)/mL.

### Cells and Virus Quantification

Madin–Darby canine kidney (MDCK) cells were cultured in a 96-well plate, with each well containing 100 µL of minimum essential medium (MEM, Shimadu Diagnostics Corporation, Tokyo, Japan) supplemented with 10% bovine fetal serum (Merck KGaA, Darmstadt, Germany), 100 U/mL penicillin G (Meiji Seika Pharma, Tokyo, Japan), 0.3 mg/mL L-glutamine (Nacalai Tesque, Kyoto, Japan), 0.1 mg/mL streptomycin (Meiji Seika Pharma), and 8 mg/mL gentamicin (MSD, Tokyo, Japan). Once the MCDK cells reached 90% confluency, each virus sample was diluted 10-fold with MEM and inoculated onto the cells, which were incubated at 37 °C in a 5% CO_2_ incubator for 3 days to observe cytopathic effects in the infected cells. Using the Reed and Muench method, virus infectivity was quantified as TCID_50_ (Reed & Muench, 1938).

### Experimental Setup for the Efficacy of Gaseous ClO_2_

**Fig. 1:**
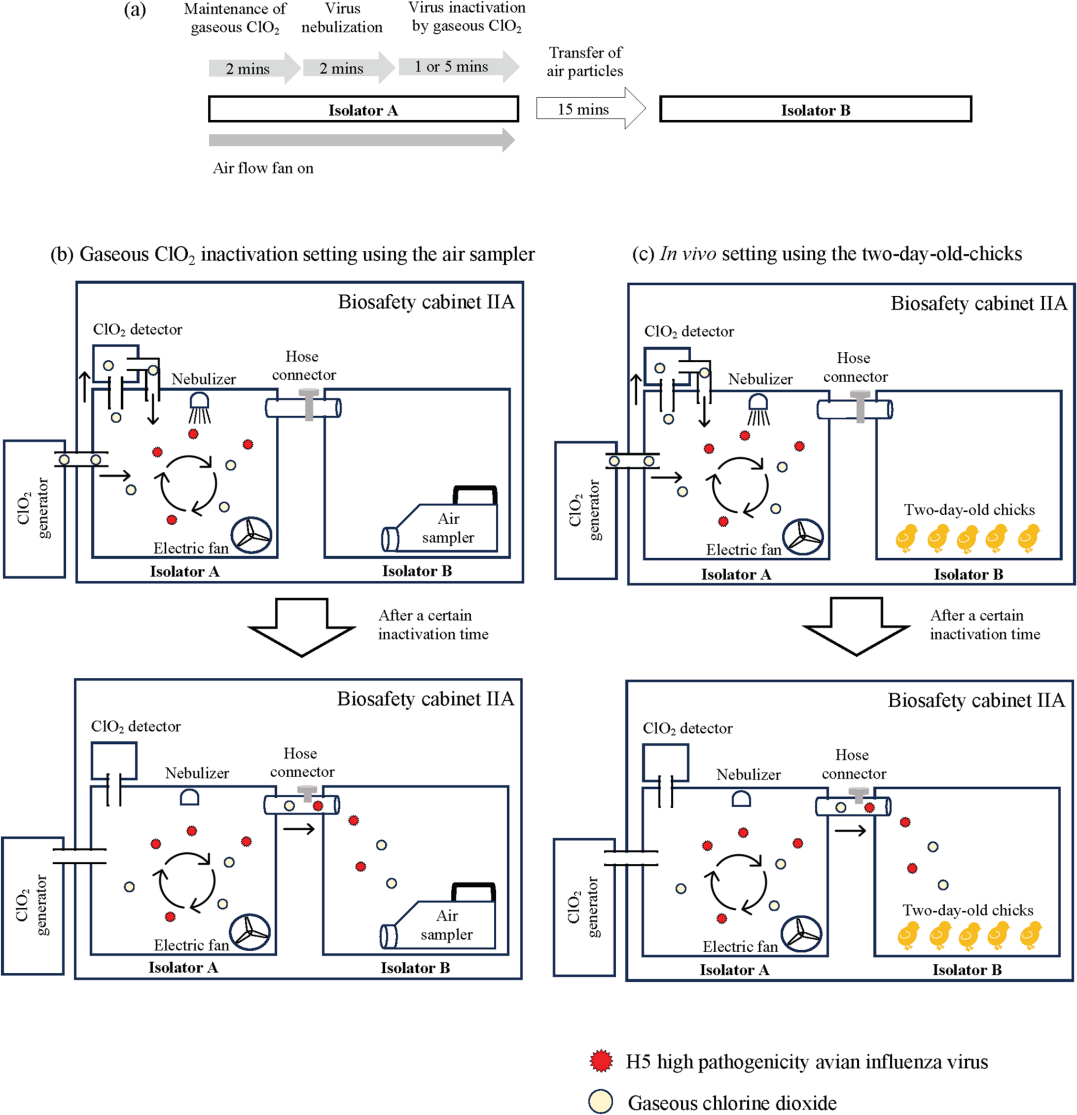
**a** Workflow of the experimental setup for inactivating aerosolized H5 high pathogenicity avian influenza virus (HPAIV) using gaseous ClO_2_. **b**, **c** The experimental setup used to evaluate the effectiveness of gaseous ClO_2_ under aerosolized H5 HPAIV conditions (**b**) and in vivo (**c**)

Two isolators, designated as isolators A and B, with dimensions of 46 × 40 × 26 cm^3^ (47.8 L) were used in this experimental setting within the class IIA biosafety cabinet (Fig. 1). The two isolators were connected by a polyvinyl chloride tube with a radius of 5 cm. Gaseous ClO_2_, produced by an ClO_2_ generator (elecloorer^®^ SS, Fujicom Co. Ltd, Aichi, Japan), or negative-control air (0 ppmv ClO_2_) was introduced into isolator A through an additional opening. A battery-powered fan was installed inside isolator A to improve air circulation, whereas nebulizers (Omron Co., Kyoto, Japan) were employed to generate aerosol from a suspension of phosphate-buffered saline or viruses suspended in sterilized distilled water. Following the interaction of the delivered gas with the nebulized viruses, the air mixture was expelled by an oil air pump into isolator B. The MD8 air sampler (Sartorius, Gottingen, Germany) collected airborne microorganisms by suctioning a specific air volume through a gelatin membrane filter (Sartorius) installed in isolator B. A sampling tube for ClO_2_ measurement was inserted into isolator A to concurrently measure and monitor the gaseous ClO_2_ concentration using a ClO_2_ gas detector (New Cosmos Electric Co., Ltd., Osaka, Japan). The concentration of gaseous ClO_2_ inside isolator A was manually controlled to 0.01 (the lowest limit of the ClO_2_ generator), 0.025, 0.05, or 0.1 ppmv by adjusting the gas supply from the ClO_2_ generator while monitoring the concentration with the ClO_2_ gas detector.

The inactivation efficacy of gaseous ClO_2_ and in vivo experiments were evaluated under the following conditions. A specific concentration of gaseous ClO_2_ (0.01, 0.025, 0.05, or 0.1 ppmv) was introduced for 2 min to maintain the gaseous ClO_2_ levels in isolator A. After nebulizing a certain concentration of viruses for 2 min, the gaseous ClO_2_ concentration in isolator A was maintained at the target level for a designated inactivation time (1–5 min, Fig. 1a). The air mixture was pumped from isolator A to isolator B by the air pump, and the air introduced into isolator B was collected by the air sampler at each sampling interval for subsequent infectivity virus quantification by TCID_50_ (Fig. 1b). For in vivo setting, chicks in isolator B were exposed to the introduced air for each exposure duration (Fig. 1c). In all experiments, temperature and relative humidity were controlled within the ranges of 22–27 °C and 62–98%, respectively.

### Evaluation of the Efficacy of Gaseous ClO_2_ Against Aerosol H5 HPAIV

Viruses from various air mixture conditions were collected using a gelatin membrane filter from the air sampler located in isolator B at a sampling velocity of 20 L/min. The filter was dissolved in 10 mL of a transport medium containing MEM containing 10 mg/mL streptomycin, 0.3 mg/mL gentamicin (MSD), 10,000 U/mL penicillin G, 0.5% bovine serum albumin fraction V (Roche, Basel, Switzerland), and 250 U/mL nystatin (Sigma-Aldrich, St. Louis, MO, USA). Then, the infectivity of the virus collected on the filter in MDCK cells under each experimental condition was expressed as TCID_50_/mL. The effectiveness of gaseous ClO_2_ against H5 HPAIV was evaluated by quantifying viral inactivation using the TCID_50_ method. Experiments were conducted independently in five rounds, primarily on different days, and viral inactivation was expressed as a log_10_ reduction, calculated as the difference between the virus titer in the absence of gaseous ClO_2_ (0 ppmv) and the titer after exposure to gaseous ClO_2_ at a specified concentration for a specified time.

### Evaluation of the Sensitivity of Chicks To H5 HPAIV

The sensitivity of chicks to H5 HPAIV was evaluated according to the 50% chicken lethal dose (CLD_50_). Two-day-old white leghorn chicks were purchased from Iwamura Poultry (Niigata, Japan) in Japan, randomly assigned into five groups (five chicks per group), and intranasally inoculated with 30 µL of WTE/Hok/R22/22 (H5N1). The infectious dosage ranged from 10^0.0^ to 10^5.0^ TCID_50_, and the mortality rate of the chicks was monitored for 14 days post-challenge (dpc).

### Evaluation of the Efficacy of Gaseous ClO_2_ Against H5 HPAIV in 2-Day-Old Chicks

Five 2-day-old white leghorn chicks purchased from Iwamura Poultry were placed in isolator B and exposed to the air mixture from each experimental condition. After disconnecting all tubes from isolator B, the clinical condition of the chicks was monitored daily for 14 dpc.

### Antibody Detection in the Chicks 14 Days After Virus Nebulization

Serum was extracted from the blood of chicks that survived 14 dpc and subjected to an HA inhibition (HI) assay to determine the antibody titer against WTE/Hok/R22/22 (H5N1). The virus was mixed with serial dilutions of serum from each surviving chick and incubated for 30 min at 37 °C, followed by the addition of 0.5% chicken erythrocytes, which were mixed for 30 min at room temperature. The HI antibody titers were expressed as the lowest concentrations of antibodies capable of completely inhibiting four HA units of the virus.

## Ethic Statement

All animal experiments were conducted at the Animal BSL-3 Facility, Faculty of Veterinary Medicine, Hokkaido University, which has been accredited by the AAALAC International since 2007. The Institutional Animal Care and Use Committee of the Faculty of Veterinary Medicine, Hokkaido University approved the experiments (approval number: 23–0121).

## Results

### Efficacy of Gaseous ClO_2_ Against Aerosol H5 HPAIV

In isolator A, gaseous ClO_2_ or air produced by the gaseous ClO_2_ generator was allowed to mix evenly with aerosolized H5 HPAIV generated by the nebulizer at an infectious dose of 10^8.0^ TCID_50_/mL, a significantly higher virus titer than typically used in experimental conditions, as described in Table 1. To facilitate virus nebulization, the original viral stock was diluted 10-fold with sterilized distilled water, as the allantoic fluid-derived stocks contain abundant proteins that could hinder the nebulizing process. In the absence of gaseous ClO_2_, the virus particles collected by the air sampler placed in isolator B exhibited virus recovery with mean titers of 2.8 ± 0.3 and 3.3 ± 0.9 log_10_ TCID_50_/mL after 0 and 5 min of inactivation, respectively. For this reason, the 5 min inactivation period, which yielded the lowest dispersion of infectious virus particles, was considered suitable for assessing the effectiveness of gaseous ClO_2_ against H5 HPAIV at an infectious dose of 10^8.0^ TCID_50_/mL. To evaluate the inactivation efficacy of gaseous ClO_2_, virus aerosols were mixed with gaseous ClO_2_ at a concentration of 0.1 ppmv for an inactivation time of 5 min. No infectious viruses were recovered by the air sampler, demonstrating the inactivation efficacy of gaseous ClO_2_ against H5 HPAIV.

**Table 1:** Infectivity of the virus recovered by the air sampler after treatment with gaseous Chlorine dioxide

Input dose (log_10_ TCID_50_/mL)	ClO_2_ (ppmv)	Inactivation time (min)	Sampling time (min)	Recovery dose (log_10_ TCID_50_/mL)	Temperature (℃)	Relative humidity (%)
8.0	0	0	10	2.8 ± 0.3	23.4 ± 2.6	64.3 ± 8.9
8.0	0	5	10	3.3 ± 0.9	25.2 ± 1.9	81.6 ± 5.9
8.0	0.1	5	10	< 0.5 ± 0.0^1^	26.1 ± 2.2	77.9 ± 4.5

^1^< 0.5 log_10_ TCID_50_/mL is the lowest detection limit of the virus titers

### Minimum Conditions Required for the Efficacy of Gaseous ClO_2_ Against Aerosol H5 HPAIV

The minimum conditions required for gaseous ClO_2_ to be effective against H5 HPAIV were evaluated using the same experimental setup. Different concentrations of gaseous ClO_2_ (0.01, 0.025, 0.05, and 0.1 ppmv) and two inactivation times (1 and 5 min) were tested. Under different gas concentration settings, the measured concentration of gaseous ClO_2_ was 0.013 ± 0.005, 0.027 ± 0.006, 0.052 ± 0.003, and 0.104 ± 0.005 ppmv. Variances in virus recovery titers observed under identical experimental conditions could be attributed to the evaluation of gaseous ClO_2_ inactivation setting being conducted on different days (rounds). Nevertheless, the overall log_10_ reduction observed under each round was consistent across all outcomes. To address this variability, all subsequent results are presented as individual values from three or more independent replicate experiments rather than as mean titers. In the absence of gaseous ClO_2_, virus titers of 2.7, 4.5, and 4.5 log_10_ TCID_50_/mL were recovered after 1 min inactivation, while titers of 3.3, 2.5, 2.7, and 4.5 log_10_ TCID_50_/mL were recovered after 5 min inactivation, using the air sampler at an infectious dose of 10^8.0^ TCID_50_/mL (Table 2). In contrast, when viruses were exposed to 0.1 ppmv of gaseous ClO_2_, a marked decrease in virus recovery was observed, with only < 0.5, 1.0, and 1.5 log_10_ TCID_50_/mL (2.2–3.5 log_10_ reduction) of infectious particles recovered after 1 min inactivation, and < 0.5 log_10_ TCID_50_/mL (2.0–2.8 log_10_ reduction) after 5 min inactivation. As the concentration of gaseous ClO_2_ decreased, titers of virus recovery increased accordingly. At 0.05 ppmv, virus titers of < 0.5, 2.0, and 2.0 log_10_ TCID_50_/mL (2.2–2.5 log_10_ reduction) and < 0.5, < 0.5, and 1.5 log_10_ TCID_50_/mL (2.0–3.0 log_10_ reduction) were confirmed after 1 and 5 min inactivation, respectively. Further reductions in gaseous ClO_2_ levels resulted in increased titers of viral recovery. At 0.025 ppmv, viruses with titers of < 0.5, 3.5, and 2.5 log_10_ TCID_50_/mL (1.0–2.2 log_10_ reduction), as well as < 0.5, < 0.5, and 2.3 log_10_ TCID_50_/mL (2.0–2.2 log_10_ reduction), were recovered after 1 and 5 min inactivation, respectively. Similarly, at 0.01 ppmv, viruses with titers of < 0.5, 3.5, and 3.3 log_10_ TCID_50_/mL (1.0–2.2 log_10_ reduction) after 1 min inactivation and < 0.5, 2.5, and 2.5 log_10_ TCID_50_/mL (2.0–2.2 log_10_ reduction) after 5 min inactivation were recovered, respectively.

**Table 2:** Assessment of minimum conditions for the efficacy of gaseous Chlorine dioxide against H5 high pathogenicity avian influenza virus

Input dose (log_10_ TCID_50_/mL)	ClO_2_ (ppmv)	Inactivation time (min)	Sampling time (min)	Recovery dose in each round (log_10_ TCID_50_/mL)					Log_10_ reduction from no gaseous ClO_2_ in each round (Log_10_ TCID_50_/mL)					Temperature (℃)	Relative humidity (%)
				1^1^	2	3	4^2^	5	1	2	3	4	5		
8.0	0	1	10	NT^3^	NT	2.7	4.5	4.5	NA^4^	NA	NA	NA	NA	24.8 ± 1.9	70.7 ± 4.9
8.0	0	5	10	3.3	2.5	2.7	4.5	-^5^	NA	NA	NA	NA	NA	25.2 ± 1.9	81.6 ± 5.9
8.0	0.01	1	10	NT	NT	< 0.5^6^	3.5	3.3	NT	NT	> 2.2	1.0	1.2	26.4 ± 0.6	67.8 ± 3.0
8.0	0.01	5	10	NT	NT	< 0.5	2.5	2.5	NT	NT	> 2.2	2.0	2.0	26.8 ± 0.9	67.3 ± 9.0
8.0	0.025	1	10	NT	NT	< 0.5	3.5	2.5	NT	NT	> 2.2	1.0	2.0	26.5 ± 0.6	62.4 ± 6.8
8.0	0.025	5	10	NT	< 0.5	< 0.5	2.3	-	NT	> 2.0	> 2.2	2.2	-	26.8 ± 0.9	63.7 ± 11.7
8.0	0.05	1	10	NT	NT	< 0.5	2.0	2.0	NT	NT	> 2.2	2.5	2.5	26.9 ± 0.2	63.9 ± 4.9
8.0	0.05	5	10	NT	< 0.5	< 0.5	1.5	-	NT	> 2.0	> 2.2	3.0	-	26.2 ± 1.2	72.4 ± 5.8
8.0	0.1	1	10	NT	NT	< 0.5	1.0	1.5	NT	NT	> 2.2	3.5	3.0	26.1 ± 0.9	64.0 ± 6.5
8.0	0.1	5	10	< 0.5	< 0.5	< 0.5	NT	NT	> 2.8	> 2.0	> 2.2	NT	NT	26.1 ± 2.2	77.9 ± 4.5

^1^Number indicates the order of independent rounds

^2^Rounds 4 and 5 were performed on the same day

^3^*NT* Not tested

^4^*NA* Not applicable

^5^-: Indicates that virus titers in Round 4 applied to Round 5, as both rounds were conducted under identical conditions on the same day

^6^< 0.5 log_10_ TCID_50_/mL is the lowest detection limit of the virus titers

### Infectivity of WTE/Hok/R22/22 (H5N1) in Chicks

To estimate the CLD_50_ of WTE/Hok/R22/22 (H5N1) in chicks, various doses of the virus were intranasally inoculated into 2-day-old chicks, which were then monitored for 14 days (Table 3). Chicks challenged intranasally with the virus at 10^4.0^ and 10^5.0^ TCID_50_/0.03 mL died within 2 dpc (Table 3). Meanwhile, four of five chicks died within 3 dpc when challenged intranasally with 10^2.0^ or 10^3.0^ TCID_50_/0.03 mL. The remaining chicks challenged with 10^0.0^–10^1.0^ TCID_50_/0.03 mL survived for 14 dpc. Based on these observations, the CLD_50_ of the chicks to H5 HPAIV was estimated to be 10^2.5^ TCID_50_.

**Table 3:** Determination of the 50% chicken lethal dose by challenge with A/white-tailed eagle/Hokkaido/22-RU-WTE-2/2022 (H5N1) in chicks

Infectious dose (log_10_ TCID_50_)	Number of surviving chicks (*n* = 5)
0.0	5
1.0	5
2.0	1
3.0	1
4.0	0
5.0	0

### Minimum Conditions for the Efficacy of Gaseous ClO_2_ Against H5 HPAIV In Vivo

To determine the minimum conditions for the efficacy of gaseous ClO_2_ against H5 HPAI in vivo setting, the same experimental setup was used to evaluate its inactivation efficacy. The measured concentrations of gaseous ClO_2_ under these conditions were 0.012 ± 0.002, 0.027 ± 0.003, 0.054 ± 0.003, and 0.104 ± 0.003 ppmv. All chicks died when exposed to aerosolized H5 HPAIV at 10^8.0^ TCID_50_/mL in the absence of gaseous ClO_2_ (Table 4). However, all chicks survived exposure to aerosolized H5 HPAIV that had been treated with 0.1 ppmv gaseous ClO_2_ for 1–5 min, consistent with the results observed in the gaseous ClO_2_ inactivation setting. Nevertheless, different outcomes emerged when the gaseous ClO_2_ concentration was reduced to 0.05, 0.025, or 0.01 ppmv. Gaseous ClO_2_ at 0.05 ppmv protected most chicks against aerosolized H5 HPAIV, with four of five chicks surviving after 5 min of inactivation, whereas only one chick survived when the inactivation time was 1 min. Conversely, only one and two chicks survived when the inactivation time was 5 min for ClO_2_ concentrations of 0.025 and 0.01 ppmv, respectively, whereas no chicks survived when the inactivation time was 1 min. No antibody against H5 HPAIV was detected in the surviving chicks by the HI test after 14 dpc. Thus, the minimum concentration (0.05 ppmv) of gaseous ClO_2_ at an inactivation time of 5 min can quickly protect against H5 HPAIV infection in chicks.

**Table 4:** Assessment of minimum conditions for the efficacy of gaseous Chlorine dioxide against H5 high pathogenicity avian influenza virus in vivo setting

Input dose (log_10_ TCID_50_/mL)	ClO_2_ (ppmv)	Inactivation time (min)	Sampling time (min)	Number of surviving chicks (*n* = 5)	HI^1^ titer	Temperature (℃)	Relative humidity (%)
8.0	0	1	10	0	NA^2^	23.7 ± 0.0	73.8 ± 2.6
8.0	0	5	10	0	NA	23.8 ± 0.1	86.1 ± 3.5
8.0	0.01	1	10	0	NA	23.7 ± 0.1	85.6 ± 1.1
8.0	0.01	5	10	2	< 2^3^	25.8 ± 0.1	98.1 ± 0.8
8.0	0.025	1	10	0	NA	24.9 ± 0.1	84.2 ± 1.5
8.0	0.025	5	10	1	< 2	24.0 ± 0.0	90.8 ± 3.0
8.0	0.05	1	10	1	< 2	25.6 ± 0.0	82.0 ± 1.6
8.0	0.05	5	10	4	< 2	24.4 ± 0.0	89.8 ± 3.9
8.0	0.1	1	10	5	< 2	23.4 ± 0.0	78.6 ± 2.1
8.0	0.1	5	10	5	< 2	22.8 ± 0.2	87.7 ± 4.5

^1^Antiserum was collected from surviving chicks 14 dpc to detect the presence of antibodies against H5 HPAIV

^2^*NA* Not applicable

^3^Average hemagglutination inhibition (HI) titer collected from the surviving chicks

## Discussion

Biosecurity measures remain a top priority for preventing avian influenza outbreaks in the poultry industry. However, even with intensive implementation of these measures, HPAI outbreaks can still occur in poultry. Therefore, effective and safe disinfectants represent additional options to halt the spread of pathogens because of their ability to decontaminate poultry farms, markets, and the surrounding environment, including surfaces and air, thereby supplementing biosecurity efforts. This study demonstrated that gaseous ClO_2_ at 0.1 ppmv can inactivate aerosolized H5 HPAIV within 1 to 5 min, achieving approximately 2.0–3.5 log_10_ reduction. Notably, a lower concentration of gaseous ClO_2_ (0.01 ppmv) with 5 min inactivation can also effectively inactivate aerosolized H5 HPAIV, achieving 2.0–2.2 log_10_ reduction, in contrast to studies evaluating the effects of gaseous ClO_2_ on various IAV strains. Numerous studies highlighted the effectiveness of gaseous ClO_2_ in suppressing multiple IAV strains, such as H1N1, achieving approximately 0.77 and 1.43 log_10_ reductions at concentrations of 0.02 and 0.1 ppmv, respectively, over 10 min (Imoto et al., 2025). Additionally, significant effects were noted against H7N9 at gaseous ClO_2_ concentrations exceeding 5.0 ppmv for 1 h of exposure (Sun et al., 2022). The difference in the effectiveness of gaseous ClO_2_ against IAVs might be attributed to experimental settings, such as humidity, the presence of organic matter, and temperature, which must be considered. In the current in vivo study, chicks, known to be the most sensitive animals to H5 HPAIV, were used as an animal model to assess the efficacy of gaseous ClO_2_ against H5 HPAIV. According to the survival of chicks challenged with different dosages of the virus, the CLD_50_ was calculated as 10^2.5^ TCID_50_, as shown in Table 3. In comparison, the CLD_50_ of a viral strain genetically similar to WTE/Hok/R22/22 (H5N1) was estimated to be approximately 10^4.5^ 50% egg infectious dose (EID_50_) in 6-week-old chickens (Isoda et al., 2022), indicating that younger chicks are more susceptible than older birds.

This study found that gaseous ClO_2_ at 0.1 ppmv, whether used for 1–5 min, can completely protect chicks against aerosolized H5 HPAIV infection. In the meantime, the minimum effective concentration of gaseous ClO_2_ against aerosolized H5 HPAIV was 0.05 ppmv for 5 min of inactivation, as most chicks survived exposure to viruses treated with this concentration of gaseous ClO_2_ for 5 min. Conversely, chicks exposed to H5 HPAIV without gaseous ClO_2_ treatment succumbed to viral infection, as demonstrated in Table 4. The clear distinction in chick mortality between the presence and absence of gaseous ClO_2_ highlights its high efficacy in inactivating H5 HPAIV in the atmosphere. This finding aligns with the virus recovery results observed in the gaseous ClO_2_ inactivation setting, as lower amounts of virus were detected in the air sample after exposure to 0.1 or 0.05 ppmv ClO_2_ for 5 min, as shown in Table 2. In addition to in vivo results, such as chick mortality rates that closely matched the virus reduction observed in the gaseous ClO_2_ inactivation efficacy, chick mortality increased as the concentration of gaseous ClO_2_ decreased. Based on the inactivation efficacy of gaseous ClO_2_, the virus recovery at 0.1 ppmv (1 and 5 min) and 0.05 ppmv (5 min) ranged < 0.5–1.5 log_10_ TCID_50_/mL, remaining insufficient to infect the chicks since the CLD_50_ is approximately 2.5 log_10_ TCID_50_. However, as the concentration of gaseous ClO_2_ declined, chick mortality increased, as observed at 0.05 ppmv (1 min) and 0.010–0.025 ppmv (1 and 5 min), where the virus recovery in the gaseous ClO_2_ inactivation setting ranged from < 0.5–3.5 log_10_ TCID_50_/mL. Although some discrepancies were noted between the inactivation efficacy of gaseous ClO_2_ and in vivo results, these are likely due to host-related and experimental factors. In vivo, variability in host susceptibility can lead to different mortality outcomes even within the same species. In addition, differences in sensitivity between the cell culture system used for viral quantification and in vivo settings may also have contributed to the varying susceptibility observed. For example, a study by Al-Dalawi et al. reported that AIV replicates more efficiently *in ovo* setting than in vitro setting (Al-Dalawi et al., 2025), indicating that different infection models can exhibit different sensitivities and may therefore yield results that do not fully align with in vitro findings. Conversely, the gaseous ClO_2_ inactivation setting used in the present study may have facilitated more effective interactions between gaseous ClO_2_ and viral particles, potentially due to better mixing during the vacuuming process via air sampling in isolator B. In contrast, in vivo setup exposed chicks only to the mixed air delivered from isolator A, which may have reduced the efficiency of viral inactivation. Nonetheless, despite these differences between experimental models, the overall trends observed in both settings were consistent with the conclusions of the study.

In prior research, viral concentrations in the air of 10^4.3^–10^6.4^ RNA copies/m^3^ were detected during an H5N8 HPAI outbreak on a poultry farm (Scoizec et al., 2018). Given that exposure to 10^3.8^–10^4.7^ EID_50_ H5 HPAIVs is sufficient to cause death in chickens (Takadate et al., 2023), 2.7 log_10_ reduction (10^6.4^ to approximately 10^3.7^ RNA copies/m^3^) in the infectivity of H5 HPAIV in the atmosphere is necessary to protect birds from HPAI on farms. 3.0 log_10_ reduction was obtained based on virus titers above the detection limit (1.5 log_10_ TCID_50_/mL) following exposure to 0.1 ppmv gaseous ClO_2_ for 1 min, as shown in Table 2. Therefore, this can be achieved using gaseous ClO_2_ at 0.1 ppmv for 1 min. However, this estimate should be interpreted with caution, as it assumes a direct relationship between RNA copies and infectious particles and it does not consider host inhalation patterns or viral deposition in the respiratory system. Regardless, it offers a helpful benchmark for assessing the effectiveness of disinfection methods. When comparing the antimicrobial efficacy of various air disinfectants, gaseous ClO_2_ also exhibited antimicrobial activity against SARS-CoV-2 in the atmosphere, achieving a 100-fold reduction within 10 min at 1.0 ppmv (Imoto et al., 2025). Similar levels of infectivity reduction were observed in SARS-CoV-2 following treatment with ozone at 1.0 ppmv (2 mg/m^3^) over the same timeframe (Imoto et al., 2025). Moreover, the infectivity of aerosolized IAV was reduced by 4-fold after exposure to 1.70 ppmv ozone at 76% relative humidity for 80 min. However, a major drawback of ozone as an air disinfectant is that its effective concentration exceeds the threshold limit (0.1 ppmv), necessitating decontamination in leak-proof rooms or the absence of humans for 80 min during its use (Dubuis et al., 2021). Contrarily, the gaseous ClO_2_ concentrations (0.1 ppmv) assessed in this study met the permissible exposure limits for public health set by the Occupational Safety and Health Administration of the United States Department of Labor (Occupational Safety and Health Administration, 1978). Furthermore, this study found that gaseous ClO_2_ was effective against viruses even at concentrations as low as 0.01 ppmv, indicating that such low and safe levels are ideal for public areas.

The efficacy of gaseous ClO_2_ in inactivating aerosolized H5 HPAIV aerosol was demonstrated in this study, providing an additional option for disinfecting aerosolized H5 HPAIV in the atmosphere and reducing viral contamination in the farm environment, thereby preventing HPAI outbreaks. In addition, the safety of gaseous ClO_2_ at 0.1 ppmv was demonstrated by the survival of chicks for 14 days with no clinical symptoms after 15 min of exposure in the same isolator (data not shown). The lack of adverse effects observed in 2-day-old chicks, which are likely more sensitive to chemical toxicity than adult birds, suggests that both chicks and chickens can generally tolerate 0.1 ppmv gaseous ClO_2_. Because 0.1 ppmv gaseous ClO_2_ is also recognized as an acceptable level for humans, applying gaseous ClO_2_ at this concentration to activate H5 HPAIVs in environments in which humans are present or chickens live, such as inside chicken farm barns, is a viable strategy. Some studies revealed the possibility of windborne transmission of HPAIV between commercial poultry outbreaks, and the tunnel ventilation system might have played a role in amplifying the infection risk (Bossers et al., 2024; Nagy et al., 2025). Outside open-type chicken farms, the airflow within farms is well-controlled by ventilation systems, which introduce outside air through ventilation ducts. In such farms, spraying gaseous ClO_2_ in front of the duct effectively reduces the risk of potential HPAIV invasion. Although constant spraying is required, the very short time needed for gaseous ClO_2_ to inactivate viruses might not necessitate spraying it throughout the entire atmosphere of the farm; instead, spraying ClO_2_ at the duct should be sufficient. Positive data on H5 HPAIV inactivation by gaseous ClO_2_ under experimental conditions should be further applied to field settings, including poultry farms.

**Fig. 2:**
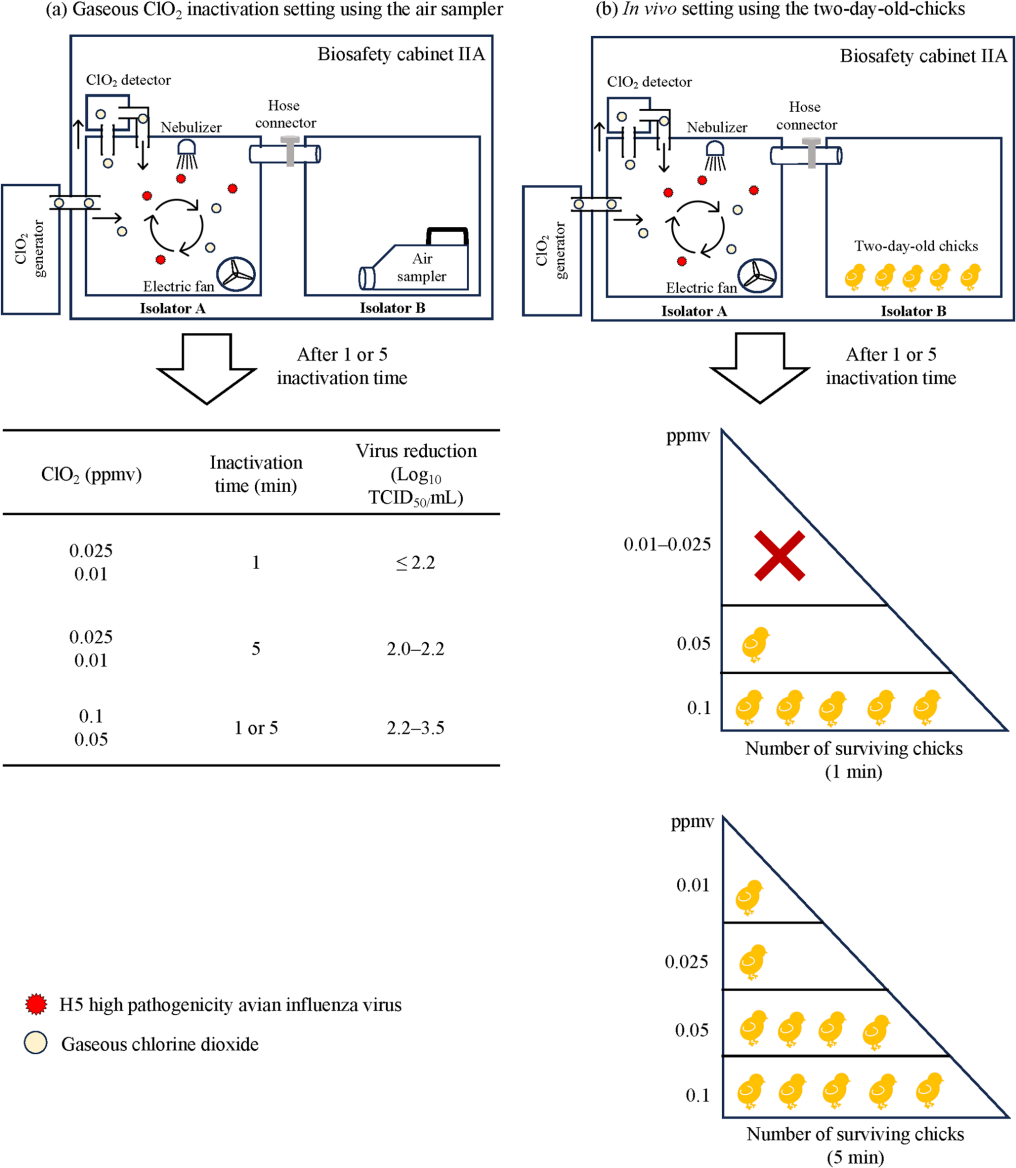
Summary of the inactivation efficacy of gaseous chlorine dioxide (ClO_2_) against aerosolized H5 high pathogenicity avian influenza virus in a gaseous ClO_2_ exposure system (**a**) and in vivo model (**b**)

This study had some limitations, as the efficacy of gaseous ClO_2_ might be influenced by the presence of organic matter. Generally, the effect of humidity on virucidal gas is an important consideration for evaluating virucidal efficacy. Similar to other gases, the virucidal effect of gaseous ClO_2_ can be affected by humidity, as humidity also includes organic matter. In the present study, as the aerosolized virus and gaseous ClO_2_ were sprayed in an airtight small isolator, humidity was maintained mostly in the range of 62–98% during the inactivation of HPAIV. This humidity level is generally higher than that in environments in which humans and chickens comfortably reside, indicating that the virucidal effect observed in this study might be overestimated compared with its effectiveness under field conditions. Although humidity was not the focus of this study, future study should examine gaseous ClO_2_ efficacy under conditions that better reflect field environments. Even at lower gaseous ClO_2_ concentrations such as 0.01 ppmv, 1 min of inactivation resulted in a 1.0–2.2 log_10_ reduction, still implying partial inactivation, whereas 5 min of inactivation resulted in a 2.0–2.2 log_10_ reduction, indicating effective inactivation. Therefore, extending exposure time or improving mixing efficiency may further enhance efficacy under low-dose conditions and facilitate easier maintenance in large, open environments. However, the atmospheric temperature should also be considered as it could also impact the effectiveness of gaseous ClO_2_ (Fukuma et al., 2025; Park & Kang., 2018). The present study demonstrated the virucidal effect of gaseous ClO_2_ against H5 HPAIV, which was confirmed for the first time, its efficacy in both gaseous ClO_2_ inactivation setting and in vivo model, as summarized in Fig. 2. The concentration of H5 HPAIV suppressed by gaseous ClO_2_ in the experimental conditions was insufficient to infect 2-day-old chicks, providing the first indication that gaseous ClO_2_ can protect chickens from HPAIV infection, and this strategy could be applied to enhance biosecurity measures in poultry. Future research should focus on testing gaseous ClO_2_ in farms and homes to assess its practical use for improving sanitation and enhancing biosecurity.

## Data Availability

The data that support this study are available from the corresponding author upon reasonable request.
